# Incremental Cost and Length of Stay Associated With Complications of Transcatheter Aortic Valve Replacement

**DOI:** 10.1016/j.jacadv.2025.102107

**Published:** 2025-08-26

**Authors:** Steven D. Culler, Matthew R. Reynolds, Aaron D. Kugelmass, Marc R. Katz, April W. Simon, David J. Cohen

**Affiliations:** a Rollins School of Public Health, Emory University, Atlanta, Georgia, USA; b Department of Economics and Quality of Life Research, Baim Institute for Clinical Research, Boston, Massachusetts, USA; c Department of Cardiology, Deborah Heart and Lung Center, Browns Mill, New Jersey, USA; d Department of Cardiothoracic Surgery, Medical University of South Carolina, Charleston, South Carolina, USA; e AWS Research, LLC, Seneca, South Carolina, USA; f Department of Clinical and Outcomes Research, Cardiovascular Research Foundation, New York, New York, USA; g Department of Academic Affairs, St. Francis Hospital, Roslyn, New York, USA

**Keywords:** aortic stenosis, complications, cost, transcatheter aortic valve replacement

## Abstract

**Background:**

Transcatheter aortic valve replacement (TAVR) has become the leading form of aortic valve replacement, with nearly 100,000 annual procedures in the United States. However, the impact of complications on hospital costs is not well understood.

**Objectives:**

The purpose of this study was to quantify the additional hospital resource use and costs associated with TAVR complications.

**Methods:**

Medicare Provider Analysis and Review file from fiscal year 2020 identified 66,893 beneficiaries undergoing TAVR. International Classification of Diseases-10th-Clinical Modification diagnostic and procedure codes were used to identify complications and hospital costs were calculated using cost-to-charge ratios. Multivariable regression estimated the incremental cost and length of stay (LOS) associated with complications.

**Results:**

In 2020, 31.1% of the 66,893 Medicare beneficiaries who underwent a TAVR experienced one or more complications. The mean cost of a TAVR hospitalization was $54,988 ± $26,744 and the mean LOS was 3.1 ± 4.5 days. Patients who experience any of the complications increased costs by $15,377 and incremental LOS by 2.8 days compared to those not experiencing complications. After adjustments for patient risk factors, the incremental cost of complications was $12,953. Surgical aortic valve replacement ($42,924), acute renal failure requiring dialysis ($34,606), and in-hospital mortality ($25,307) had the highest risk-adjusted costs. Complications accounted for $320 million in additional costs, representing 8.7% of the total hospital cost for Medicare TAVR patients.

**Conclusions:**

In contemporary practice, approximately one in 3 Medicare TAVR patients experience serious complications, substantially increasing hospital resource use. Despite improvements in TAVR outcomes and efficiency, additional cost savings can be achieved through complication mitigation.

Over the past decade, transcatheter aortic valve replacement (TAVR) has profoundly impacted the volume and outcomes of aortic valve interventions. Since the U.S. Food and Drug Administration approval of TAVR in 2011, the overall volume of aortic valve procedures has increased substantially^1^ along with a marked shift from surgical to TAVR. In 2020, approximately 80,000 isolated aortic valve procedures were performed in the Medicare population, of which more than 85% were TAVR.^1^ With continued expansion of indications for TAVR and current demographic trends, it is anticipated that the volume of TAVR (and associated Medicare expenditures) will continue to increase.

While complications associated with TAVR have decreased substantially from initial reports,^2^, ^3^, ^4^ complications still occur in approximately 30% of all TAVR patients.^1^ Prior studies have shown that arrhythmias, vascular complications, and transfusion are the 3 most common complications among patients undergoing TAVR procedures.^2^, ^3^, ^4^, ^5^, ^6^, ^7^ However, there have been relatively few studies examining the costs of TAVR complications—particularly since the approval of TAVR for low-risk patients in 2019.^2^^,^^5^ Moreover, there have been no national studies to provide benchmark statistics on the cost of complications following TAVR in current practice. To address these gaps in knowledge, we used Medicare data to estimate the incremental hospital resources consumed (cost and length of stay [LOS]) among patients that experience complications during their TAVR hospitalization and their contribution to the overall cost of TAVR in the United States.

## Methods

This retrospective study used the Medicare Provider Analysis and Review (MedPAR) administrative data set from fiscal year 2020. The MedPAR file, maintained by the Centers for Medicare and Medicaid Services, contains all inpatient claims submitted by hospitals for patients covered by either the traditional Medicare or the Medicare Advantage programs. For each hospitalization, the MedPAR record includes demographic patient information, LOS in whole days, discharge status, and hospital charges. In addition, the MedPAR files report up to 25 diagnosis and 25 procedure codes per admission using the International Classification of Diseases-10th Edition-Clinical Modification (ICD-10-CM). Because all data analyzed in this manuscript are from publicly available deidentified administrative data set, it did not need Institutional Review Board approval.

### Study population

The study population consisted of all Medicare beneficiaries who underwent a TAVR procedure during fiscal year 2020. Patients were excluded if they underwent concomitant replacement of another valve and/or coronary artery bypass grafting (CABG), were treated at a non-U.S. hospital or a low-volume TAVR center (<24 Medicare beneficiaries treated in 2020), or if cost data were missing.

### Definitions of complications

In-hospital mortality and 10 complications (selected on the basis of published studies^2^, ^3^, ^4^, ^5^ and expert opinion) were defined for this study. The complications included stroke; cardiac dysfunction (heart failure, cardiac insufficiency, or cardiac arrest associated with the TAVR); acute renal failure without dialysis; acute renal failure with dialysis; arrhythmia (heart block, atrial fibrillation or flutter, or ventricular arrhythmia) without permanent pacemaker (PPM) implantation; PPM implantation; repeat TAVR; surgical aortic valve replacement (SAVR); vascular complication (vascular repair complication, intraoperative hematoma/hemorrhage, or postoperative hematoma/hemorrhage); and transfusion. ICD-10-CM codes and present-on-admission flags were used to distinguish in-hospital complications from possible comorbidities. For complications with multiple potential ICD-10-CM codes, the complication was considered to have occurred when at least one appropriate code was listed as “not present on admission.” Appendix A lists the ICD-10-CM procedure and diagnosis codes used to identify each complication reported in this study.

### Patient characteristics and comorbidities

Patient and procedural characteristics were identified from the claims files and included sociodemographic factors (age, sex, race); admission status (elective, urgent, emergent); admission source (clinic vs emergency room vs hospital transfer); and presenting conditions (atrial fibrillation, heart failure, and respiratory failure); and prior procedures including prior valve surgery, prior CABG, prior percutaneous coronary intervention, prior PPM, and prior implantable cardioverter defibrillator. Finally, we identified comorbid conditions that have been shown to increase hospital resource utilization independent of complications. These included diabetes mellitus, malnutrition, anemia, previous myocardial infarction, prior stroke, hypertension, obstructive sleep apnea, chronic obstructive pulmonary disease, acute renal failure, chronic kidney disease, dialysis dependence, chronic liver disease, and peripheral vascular disease. TAVR access site was characterized as transfemoral, transapical, or unknown. All comorbidities were defined based on ICD-10-CM diagnosis codes contained in the MedPAR file.

### Hospital resource utilization

The key outcome measures for our study were total cost for the TAVR hospitalization and LOS. Of note, the MedPAR file used for this study includes only resources consumed during the TAVR hospital admission and does not include other health care services such as those provided by physicians or other postdischarge facilities. Hospital resource costs (in 2020 U.S. dollars) were estimated by multiplying total charges reported in the MedPAR file by the hospital’s overall cost-to-charge ratio, obtained from the fiscal year 2020 Medicare Cost Report. This method is commonly used to estimate hospital cost from claims data sets.^8^, ^9^, ^10^ For a population-based study where maximizing generalizability of cost estimates is an important goal, this approach avoids several of the estimation challenges that arise when using departmental cost-to-charge ratios including allocating overhead costs to departments, determining which hospital cost-to-charge ratio to assign to charge codes that do not match any reported departmental ratio, and the uncertainty concerning whether TAVR patients use the typical services in any department.^11^

### Statistical analysis

Differences in baseline patient characteristics were compared between patients who experienced a complication and those who did not, using chi-square analysis or Fisher exact test for discrete variables. Observed event rates are reported as the proportion of hospitalizations with a selected event among all study hospitalizations. Hospital cost and LOS are presented as mean ± SD (with selected median values). All multivariable regression models were estimated using the linear and log-linear forms of the estimated resource equation. However, we only report the linear results because the focus is on evaluating the expected cost value, as represented by the mean. This approach is justified by the robustness provided by the large sample size, and the relative ease of interpreting estimates in the linear model vs the log-linear alternative. For both the estimated cost and LOS equations, we report incremental and attributable costs and LOS. Incremental cost (or LOS) refers to the additional resources required to treat each patient who experiences a specific complication. In contrast, attributable cost (or LOS) represents the additional resources a complication adds to the expected average hospital cost (or LOS) for each patient undergoing a TAVR procedure, regardless of whether that patient actually experiences the complication. In this study, differences between study groups were considered statistically different if the *P* value was ≤0.01. All analyses were performed with SAS 9.4 (SAS Institute).

### Approach to estimating for incremental resource use

Incremental resource use associated with each complication was estimated using 2 approaches. First, we calculated the incremental cost (or LOS) as the difference between the mean cost (or LOS) among patients who experienced only the event of interest compared with the mean value among patients who did not experience any complications. In this approach, we excluded any patient who experienced more than one complication. In the second approach, we used multivariable linear regression to adjust for all the demographic, clinical, and procedural variables described above to estimate the incremental cost or LOS of each complication, while simultaneously adjusting for all other complications. Under this approach, incremental-adjusted resources (cost and LOS) associated with each complication are represented by the regression coefficient for the complication of interest.

## Results

### Patient population

**Figure 1:**
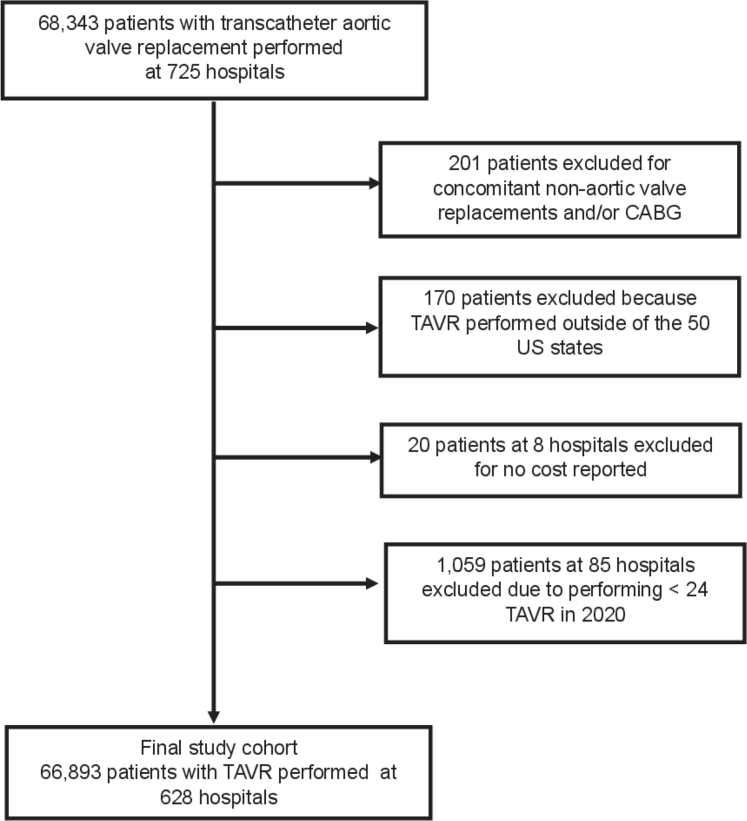
**Flow Diagram Depicting Selection of the Study Cohort From the 2020 MedPAR Files and Main Exclusion Criteria** TAVR = transcatheter aortic valve replacement.

In 2020, there were 68,343 hospital admissions for TAVR among Medicare beneficiaries. After excluding 201 hospitalizations with concomitant valve and/or CABG procedures, 170 hospitalizations in a U.S. territory, 20 hospitalizations that were missing cost data, and 1,059 hospitalizations performed in low-volume centers, the final study population consisted of 66,893 TAVR hospital admissions performed at 628 U.S. hospitals (Figure 1). Among these hospitals, mean Medicare TAVR volume was 107 ± 80 (median 82; range: 24-593). Baseline patient characteristics are summarized in Table 1. The median age of the study cohort was 80, 44% were female, 92% were White, 85% of admissions were elective, and femoral access was used in 97%.

**Table 1:** Baseline Characteristics of the Study Population, Stratified According to the Occurrence of In-Hospital Complications

	Total TAVR Population (n = 66,893)	TAVR With Complication(s) (n = 20,787)	TAVR Without Complication(s) (n = 46,106)	*P* Value
Age				<0.001
<65 y	2.2%	2.3%	2.2%	
65-69 y	10.0%	9.8%	10.1%	
70-74 y	15.7%	14.7%	16.1%	
75-79 y	21.2%	20.6%	21.5%	
80-84 y	22.9%	22.%	23.0%	
>85 y	27.9%	29.7%	27.1%	
Male	56.3%	54.0%	57.3%	<0.001
Race				
White	91.6%	91.2%	91.7%	
Non-White	8.4%	8.8%	8.3%	0.038
Admission status				<0.001
Elective	84.9%	79.0%	87.6%	
Urgent	8.7%	10.9%	7.6%	
Emergent	6.5%	10.0%	4.8%	
Source of admission				<0.001
MD office/clinic	94.2%	91.9%	95.3%	
Transfer from acute care	4.3%	6.5%	3.3%	
Transfer from postacute care facility	0.9%	1.2%	0.7%	
Other	0.6%	0.5%	0.7%	
TAVR approach				<0.001
Femoral	97.2%	97.3%	97.1%	
Nonfemoral	2.8%	2.7%	2.9%	
Presenting conditions				
Heart failure	69.6%	72.3%	68.4%	<0.001
Acute respiratory failure	2.5%	3.8%	1.9%	<0.001
Atrial fibrillation	34.8%	33.8%	35.3%	<0.001
Prior procedures				
Prior valve surgery	3.2%	2.8%	3.4%	<0.001
Prior CABG	13.3%	11.8%	14.0%	<0.001
Prior PCI	21.9%	20.1%	22.7%	<0.001
Prior PPM	9.0%	4.1%	11.2%	<0.001
Prior ICD	2.2%	1.1%	2.7%	<0.001
Cardiac comorbidities				
Atrial fibrillation	34.8%	33.8%	35.3%	<0.001
Prior MI	11.5%	11.3%	11.5%	0.518
Prior stroke	11.6%	10.9%	11.9%	<0.001
Peripheral artery disease	11.1%	12.7%	10.4%	<0.001
Noncardiac comorbidities				
Diabetes mellitus				
Malnutrition	29.0%	29.6%	28.8%	<0.001
Anemia	2.1%	3.6%	1.4%	0.518
Pulmonary hypertension	13.9%	15.8%	13.1%	<0.001
Obstructive sleep apnea	17.6%	17.6%	17.6%	0.918
COPD	19.3%	19.9%	19.0%	0.003
Chronic kidney disease	23.1%	27.0%	21.4%	<0.001
Dialysis dependent	3.4%	4.1%	3.1%	<0.001
Chronic liver disease	2.1%	2.2%	2.1%	0.240

CABG = coronary artery bypass grafting; COPD = chronic obstructive pulmonary disease; ICD = implantable cardioverter defibrillator; MD = medical doctor’s; MI = myocardial infarction; PCI = percutaneous coronary intervention; PPM = permanent pacemaker; TAVR = transcatheter aortic valve replacement.

### In-hospital complications

Among 66,893 patients undergoing TAVR, 20,787 (31.1%) experienced one or more in-hospital complications. In-hospital complications were associated with increased age, female sex, nonelective admission, transfers between facilities, and use of a nonfemoral access site (Table 1). In addition, complications were associated with higher rates of most comorbid conditions.

Table 2 provides additional details regarding the type and frequency of complications. A total of 660 (1.0%) patients died during the TAVR admission. In the overall study cohort, 24.1% (16,143) experienced only one complication, 5.4% (3,592) experienced 2 complications, 1.1% (757) experienced 3 complications, and 0.4% (295) experienced four or more complications. The most common complications were arrhythmia without pacemaker implantation (15.8%), PPM implantation with or without arrhythmia (7.5%), and transfusion (4.6%).

**Table 2:** Frequency of In-Hospital Complications

Any complication	20,787 (31.1)
Number of complications	
0	46,106 (68.9)
1	16,143 (24.1)
2	3,592 (5.4)
3	757 (1.1)
>4	295 (0.4)
Specific complications (in order of frequency)	
Arrhythmia without permanent pacemaker	10,584 (15.8)
Permanent pacemaker implantation	5,032 (7.5)
Transfusion	3,076 (4.6)
Acute renal failure without dialysis	2,653 (4.0)
Vascular complication	2,252 (3.4)
Vascular repair	1,388 (2.1)
Intraoperative hematoma/hemorrhage	214 (0.3)
Postoperative hematoma/hemorrhage	847 (1.3)
Cardiac dysfunction	1,663 (2.5)
Heart failure or insufficiency	1,576 (2.4)
Cardiac arrest	97 (0.1)
Death	660 (1.0)
Stroke	445 (0.7)
Acute renal failure with dialysis	272 (0.4)
Repeat TAVR	134 (0.2)
Surgical AVR	91 (0.1)

Values are n (%).

AVR = aortic valve replacement; other abbreviation as in Table 1.

### Cost of complications

Table 3 summarizes the observed (unadjusted) mean hospital cost and LOS along with the incremental cost and incremental LOS for patients experiencing any complication, a single complication only, and each of the 11 individual complications that occurred during the TAVR admission (as isolated complications). In the overall cohort, the mean cost of a TAVR hospitalization was $54,988 ± $26,744 (median $50,763) and the mean LOS was 3.1 ± 4.5 (median 2, IQR: 1-3) days. Compared with patients who did not experience a complication, patients who experienced one or more complications had significantly higher mean costs ($65,432 ± $36,451 vs $50,055 ± $18,977; *P* = 0.001) and significantly longer LOS (5.0 ± 6.4 days vs 2.2 ± 3.0; *P* = 0.001). Among patients who experienced only a single complication, 6 of 11 complications were associated with an incremental cost >$15,000: acute renal failure with dialysis, SAVR, death, acute renal failure without dialysis, PPM implantation, and transfusion. Each of the complications resulted in observed incremental LOS >2.0 days except for arrhythmia without PPM, cardiac dysfunction, repeat TAVR, and vascular complications. Compared with patients who experienced no complications, those who experienced multiple complications were particularly resource intensive to treat, with an observed incremental hospital cost of $26,306 and incremental LOS of 5.0 days for those patients experiencing 2 complications and $62,048 and 10.1 hospital days for those patients who experienced 3 or more complications.

**Table 3:** Unadjusted Cost and Length of Stay Associated With Specific Complications

	Cost, $	Incremental Cost, $^a^	LOS (Days)	Incremental LOS (Days)^a^
All TAVR (N = 66,893)	54,988 ± 26,744	4,933	3.1 ± 4.5 (1-3)	0.9
No complication (n = 46,106)	50,055 ± 18,977	NA	2.2 ± 3.0 (1-2)	NA
Any complication (n = 20,787)	65,432 ± 36,451	15,377	5.0 ± 6.4 (2-6)	2.8
Any single complication (n = 16,143)	60,602 ± 28,063	10,547	4.0 ± 5.2 (1-5)	1.8
Patients experiencing only 1 complication				
Death (n = 128)	82,920 ± 65,970	32,865	6.9 ± 16.4 (1-9)	4.7
Stroke (n = 222)	62,776 ± 25,501	12,721	5.5 ± 4.6 (3-7)	3.3
Cardiac dysfunction (n = 165)	57,364 ± 32,260	7,309	3.1 ± 4.3 (1-3)	0.9
Acute renal failure without dialysis (n = 983)	70,182 ± 34,242	20,127	8.0 ± 7.1 (3-11)	5.8
Acute renal failure with dialysis (n = 45)	93,951 ± 39,912	43,896	14.5 ± 9.4 (9-19)	12.3
Arrhythmia without PPM (n = 8,355)	54,243 ± 24,601	4,188	3.0 ± 4.1 (1-3)	0.8
PPM implantation (n = 3,547)	69,613 ± 27,200	19,558	4.3 ± 4.2 (2-5)	2.1
Repeat TAVR (n = 76)	60,427 ± 29,650	10,372	3.3 ± 3.5 (1-5)	1.1
Surgical AVR (n = 17)	89,432 ± 28,842	39,377	7.2 ± 3.5 (5-10)	5.0
Vascular complication (n = 1,157)	60,093 ± 23,714	10,038	4.0 ± 4.6 (1-5)	1.8
Transfusion (n = 1,448)	65,821 ± 30,304	15,766	6.1 ± 6.8 (2-8)	3.9

Values are mean ± SD or mean ± SD (1st-3rd quartile) unless otherwise indicated.

Abbreviations as in Tables 1 and 2.

a Incremental cost (LOS) refers to the difference in mean cost (LOS) between patients with and without the complication of interest.

Table 4, Table 5 present estimates of the risk-adjusted incremental cost and LOS associated with each of the complications. The risk-adjusted incremental cost of any complication was $12,953 with an incremental LOS of 2.0 days. The highest incremental cost was seen among patients who required SAVR during the hospitalization ($42,924), patients who experienced acute renal failure requiring dialysis ($34,606), and patients who died during their TAVR hospitalization ($25,307). All other complications were associated with incremental costs between $10,000 and $20,000 with the exception of cardiac dysfunction ($4,013) and arrhythmia without a new pacemaker ($4,696). The estimated incremental LOS exceeded 1 day for all study complications, except for death (0.7 days), cardiac dysfunction (0.3 days), arrhythmia without a new pacemaker (0.8 days), and repeat TAVR (0.5 days).

**Table 4:** Incremental and Attributable Hospital Costs Associated With Complications of TAVR

Complication	Incremental Cost (95% CI), $^a^	Attributable Cost, $^b^
Any complication	12,953 (12,555-13,350)	4,028
Specific complications		
Death	25,307 (23,496-27,118)	253
Stroke	11,110 (9,002-13,219)	78
Cardiac dysfunction	4,013 (2,873-5,154)	100
Acute renal failure without dialysis	18,815 (17,878-19,753)	753
Acute renal failure with dialysis	34,606 (31,795-37,417)	138
Arrhythmia without PPM	4,696 (4,212-5,180)	742
PPM implantation	18,734 (18,055-19,414)	1,405
Repeat TAVR	11,907 (8,074-15,739)	24
Surgical AVR	42,924 (38,248-47,599)	43
Vascular complication	10,536 (9,568-11,504)	358
Transfusion	11,958 (11,105-12,811)	550

Abbreviations as in Tables 1 and 2.

a Incremental cost for individual complications based on multivariable linear regression model including all baseline and procedural characteristics along with all complications. Complications are not mutually exclusive. Incremental cost refers to the predicted additional resources required to treat each patient who experiences a specific complication.

b Attributable cost is the incremental cost associated with the complication multiplied by the incidence of the complication.

**Table 5:** Incremental Length of Stay Associated With Complications of TAVR

	Incremental LOS (95% CI) in Days^a^	Attributable LOS^b^
Any complication	2.0 (1.9-2.0)	0.61
Specific complications		
Death	0.7 (0.5-1.0)	0.01
Stroke	3.4 (3.1-3.7)	0.02
Cardiac dysfunction	0.3 (0.2-0.5)	0.01
Acute renal failure without dialysis	4.5 (4-4.7)	0.18
Acute renal failure with dialysis	6.0 (5.6-6.4)	0.02
Arrhythmia without PPM	0.8 (0.7-0.8)	0.12
PPM implantation	1.8 (1.7-1.8)	0.13
Repeat TAVR	0.5 (−0.2-1.1)	0.00
Surgical AVR	3.8 (3.1-4.4)	0.00
Vascular complication	1.5 (1.4-1.7)	0.05
Transfusion	2.2 (2.1-2.4)	0.10

Abbreviations as in Tables 1 and 2.

a Incremental LOS for individual complications based on multivariable linear regression model including all baseline and procedural characteristics along with all complications. Incremental LOS refers to the predicted additional hospital days required to treat each patient who experiences a specific complication.

b Attributable LOS is the incremental LOS associated with the complication multiplied by the incidence of the complication.

### Attributable costs

Table 4 also summarizes the risk-adjusted attributable cost for each complication—that is, the additional cost that the specific complication adds to the overall in-hospital cost of TAVR in Medicare beneficiaries. The attributable cost for any complication was $4,028—roughly 7% of the overall cost of TAVR and 17% of the nondevice-related costs (assuming a cost of $30,000 for the TAVR device)—while the attributable LOS for any complication was 0.61 days. The complications with the highest attributable cost estimates were new pacemaker with or without arrhythmia ($1,405), acute renal failure without dialysis ($753), and arrhythmia without PPM ($742). On the other hand, owing to low frequency of specific complications in the Medicare population, the attributable in-hospital cost was <$100 for stroke ($78), SAVR ($43), and repeat TAVR ($24). The risk-adjusted attributable LOS was <0.2 days for each of the individual complications (Table 5, Central Illustration).

**Central Illustration:**
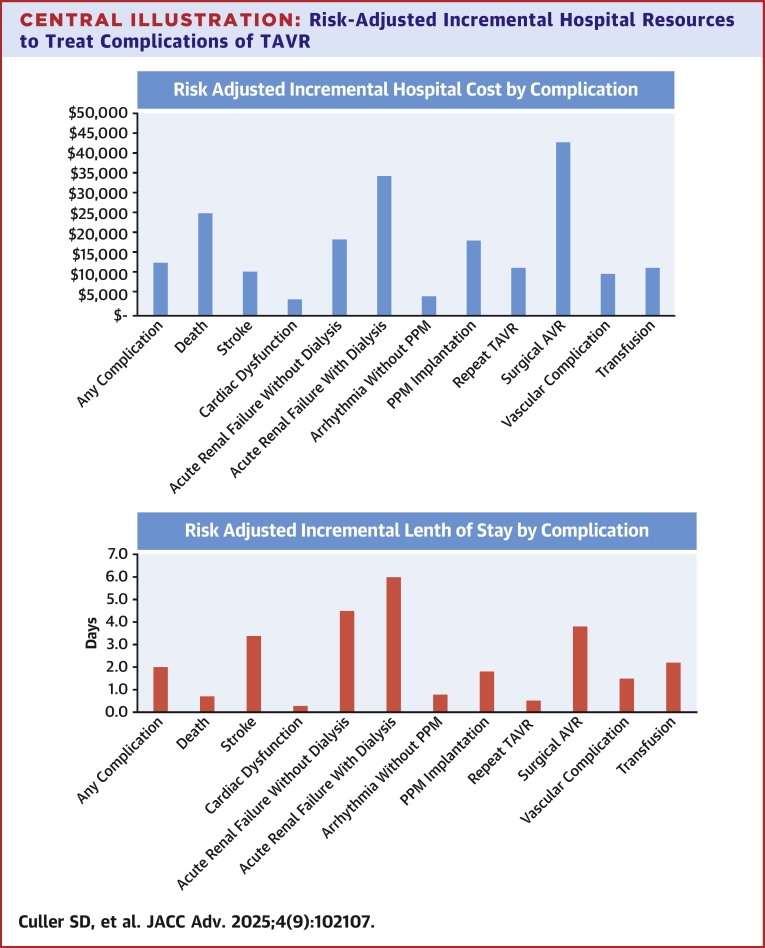
**Risk-Adjusted Incremental Hospital Resources to Treat Complications of TAVR** After risk adjusting, the incremental cost of an individual complication exceeded $15,000 for 5 complications—conversion to SAVR, acute renal failure requiring dialysis, death, acute renal failure without dialysis, and permanent pacemaker implantation. These same complications were associated with an incremental length of stay of >2 days apart from permanent pacemaker implantation. The total cost of treating all complications identified in this study accounted for approximately 8.7% of the total hospital cost of treating all Medicare beneficiaries undergoing a TAVR procedure. AVR = aortic valve replacement; PPM = permanent pacemaker; SAVR = surgical aortic valve replacement; other abbreviation as in Figure 1.

## Discussion

This study of more than 60,000 Medicare beneficiaries undergoing TAVR at 628 different hospitals using contemporary devices and techniques provides several key insights into the frequency and cost of complications during the TAVR hospitalization. First, nearly one-third of all Medicare beneficiaries experienced at least one clinically relevant complication. Although this frequency has decreased substantially over the past decade,^2^, ^3^, ^4^ these rates remain relatively high. Second, this study provides a set of nationally representative cost benchmarks for the cost of TAVR in contemporary practice as well as the cost of associated complications. For the 69% of patients who did not experience a complication, the total hospital cost was $50,055, which increased to $65,432 among patients who experienced one or more major complications. After adjusting for differences in baseline patient and procedural characteristics, the incremental cost was modestly lower but remained nearly $13,000 per patient with an incremental LOS of 2.0 days. Finally, the incremental cost of an individual complication exceeded $15,000 for 5 complications—conversion to SAVR, acute renal failure requiring dialysis, death, acute renal failure without dialysis, and PPM implantation. These same complications were associated with an incremental length of stay of >2 days with the exception of PPM implantation.

### Comparison with previous studies

Several previous studies have examined the cost of TAVR-related complications in the United States.^2^ Arnold and colleagues examined the frequency and cost of complications among patients at high surgical risk in the PARTNER (Placement of Aortic Transcatheter Valves) 1A and 1B trials. Among 406 patients, major complications were observed in 49% and were associated with an incremental cost of ∼$33,000 and an incremental LOS of 6.6 days. More recently, Okoh and colleagues reported on 1,163 patients who underwent TAVR between 2012 and 2018 at a single academic medical center. In their study, the overall frequency of complications was 18%, and the incremental cost of one or more complications was ∼$11,000.^12^ Our study adds to these previous studies by including a much larger sample size across more than 600 U.S. hospitals and also by including patients across the full surgical risk spectrum. Given the much larger sample size, our study was able to provide more precise estimates of the cost of each complication.

The most striking difference between the studies is the much lower attributable cost of complications in contemporary practice compared with the PARTNER trials^2^—decreasing from ∼$13,000/patient to ∼$4,000/patient in contemporary practice. Attributable cost, which is calculated by multiplying the incremental cost of each complication by the frequency of the complication in the overall population, represents the hypothetical contribution of all complications to the cost of the average TAVR hospitalization. This striking reduction in complication-related costs reflects both reductions in the frequency of most complications in contemporary practice as well as lower incremental cost for most complications as well. In addition, the current study demonstrates that the cost of uncomplicated TAVR has decreased substantially as well—from ∼$63,000 in 2010 to ∼$50,000 in 2020. After excluding the cost of the TAVR valve, we estimate that nondevice-related costs for an uncomplicated TAVR procedure have decreased by 39% from $33,000 to $20,000 over this time period. The main driving factor for this cost reduction is a marked reduction in mean length of stay for an uncomplicated TAVR from 7.4 days in 2010 to 2.2 days in 2020. This decrease in length of stay for uncomplicated TAVR reflects the widespread adoption of best practices over the past decade including use of moderate sedation, fewer central lines, as well as accelerated mobilization and standardized patient pathways to encourage next day discharge.^13^^,^^14^

### Clinical and economic implications

Our study has important implications for strategies to improve the efficiency and cost-effectiveness of TAVR in contemporary practice. Although the incremental cost was highest for in-hospital death, acute renal failure requiring dialysis, and conversion to SAVR, the attributable cost of each of these complications was <$300—reflecting the infrequency of each complication in the study population. In contrast, the attributable cost was much higher for more common complications such as major transfusion ($550), acute renal failure without dialysis ($753), postprocedure arrhythmia without pacemaker ($742), and new pacemaker placement ($1,405). These findings suggest that interventions that reduce postprocedure bleeding, renal failure, arrhythmias, and the need for new pacemaker after TAVR should have the greatest impact on overall hospital costs. Whether further reductions in the cost of uncomplicated TAVR can be achieved (without reducing the cost of the TAVR device, itself) is unclear as most U.S. centers have adopted “minimalist” procedural techniques along with “fast-track” TAVR pathways in recent years.

### Study Limitations

This study has several important limitations. First, our study applies only to Medicare beneficiaries. Although most patients undergoing TAVR are over age 65 (and thus Medicare eligible), the extent to which our findings on the incremental cost of complications can be extrapolated to other patient groups is unclear—especially younger patients who may be able to recover from a complication consuming fewer resources. Second, TAVR-related complications were identified based on ICD-10-CM coding rather than review of medical records as would be performed in a clinical trial or registry. This limitation is mitigated by the fact that the MedPAR file now contains 25 specific ICD-10-CM codes, which allow for more precise identification of complications, as well as present-on-admission flags to differentiate between clinical conditions that existed on admission and those that occurred during or after the procedure. Nonetheless, it is possible that miscoding of certain complications may have resulted either underestimation or overestimation of their incremental costs. Since it is likely that undercoding of complications would result in overestimation of their incremental costs (and vice versa), the net effect on attributable costs would be minimized.

Third, hospital resource costs were estimated from charges and may not measure true resources consumed. However, this approach has been validated compared with hospital accounting systems^8^ and has been used in multiple prior studies of cardiovascular procedures.^9^^,^^14^, ^15^, ^16^, ^17^, ^18^ Moreover, with over 600 different hospitals in our study, it is unlikely that our approach used to estimate cost would consistently over or underestimate the cost of treating patients in either study group. It is also reassuring that a similar pattern of incremental resource utilization emerges from our analysis of LOS data. Fourth, our estimates of the attributable cost of complications assume that each complication is independent of the others. However, if certain complications are causally linked (eg, transfusion may lead to acute renal failure; stroke may lead to death), interventions that prevent the proximate complication may lead to greater cost savings than we have estimated since the “downstream” complication could be avoided as well. On the other hand, it is possible that some complications may have been caused by events that led to the TAVR procedure—particularly among patients undergoing nonelective procedures. Nonetheless, our estimates of attributable costs still reflect the cost associated with each specific complication—regardless of its root cause. Finally, it is important to recognize that the incremental and attributable costs we identified represent costs from the hospital’s perspective and may not fully account for the cost to the health care system, especially for complications such as stroke that are associated with substantial “downstream” costs.

## Conclusions

Based on this study of Medicare claims, approximately one-third of all patients undergoing TAVR in 2020 experienced one or more major complications with a total attributable cost of ∼$4,000 per patient—accounting for approximately 17% of the nondevice-related cost of the hospitalization. When considering both the incremental cost and frequency of complications, the main contributors to the cost of complications appear to be new pacemaker placement, postprocedure arrhythmias (without pacemaker), acute renal failure without dialysis, and transfusion. These findings suggest that despite substantial improvements in TAVR outcomes and efficiency in recent years through the adoption of best practices, there remain substantial additional cost savings to Medicare and society, in general, that can be achieved through complication mitigation.

Perspectives **COMPETENCY IN PATIENT CARE**

With the continued expansion of indications, annual volumes of TAVR are approaching 100,000 procedures in the United States. Among Medicare beneficiaries in fiscal year 2020, one in 3 patients experienced a complication during their TAVR procedure.

**TRANSLATIONAL OUTLOOK 1**

This study provides national estimates for the incremental and patient-adjusted cost of eleven complications occurring during a TAVR procedure. In aggregate, treating complications accounted for $320 million of additional cost—approximately 8.7% of the total hospital cost of treating all Medicare beneficiaries undergoing a TAVR procedure.

**TRANSLATIONAL OUTLOOK 2**

This article suggests that by improving clinical outcomes and reducing complications among TAVR patients will result in substantial additional cost savings.

## Funding support and author disclosures

Dr Reynolds has received consulting fees from 10.13039/100006520Edwards Lifesciences and 10.13039/100004374Medtronic. Dr Katz has received institutional research grant support from 10.13039/100000046Abbott, 10.13039/100008497Boston Scientific, 10.13039/100006520Edwards Lifesciences, HighLife, 10.13039/100019998JenaValve, and 10.13039/100004374Medtronic; and has received consulting fees from 10.13039/100000046Abbott, 10.13039/100008497Boston Scientific, 10.13039/100006520Edwards Lifesciences, Enable CV, and 10.13039/100004374Medtronic. Dr Cohen has received institutional grant support from 10.13039/100006520Edwards Lifesciences, 10.13039/100004374Medtronic, 10.13039/100008497Boston Scientific, 10.13039/100000046Abbott, Philips, 10.13039/100019998JenaValve, Corvia, 10.13039/100015345Zoll Medical, and Cathworks; and has received consulting fees from 10.13039/100006520Edwards Lifesciences, 10.13039/100004374Medtronic, 10.13039/100008497Boston Scientific, 10.13039/100000046Abbott, and Elixir Medical. All other authors have reported that they have no relationships relevant to the contents of this paper to disclose.
