# Supplementary material for: Incremental Cost and Length of Stay Associated With Complications of Transcatheter Aortic Valve Replacement
Source: JACC Adv. 2025 Aug 26;4(9):102107. doi: 10.1016/j.jacadv.2025.102107 (PMC12409306; doi:10.1016/j.jacadv.2025.102107)
Supplement: Supplemental Material [file mmc1.docx]

**Supplemental Table 1.** ICD-10-CM Codes for Identifying Complications

| **Complications:** | **Codes** |
| --- | --- |
|  |  |
| Death | Discharge Status coded = ‘20’, ‘40’, ‘41’, or ‘42’ |
| Stroke | Dx Code = ('I97810', 'I97811', 'I97820', or 'I97821') |
| Cardiac dysfunction | Dx Code = ('I97111', 'I97130', 'I97110', 'I97131', 'I97190', 'I97191', 'I97120', or 'I97121') and POA Code NE ‘Y’ |
| ARF with dialysis | PX Code = ('5A1D60Z', '5A1D00Z', '5A1D70Z', '5A1D80Z', or '5A1D90Z') and patient was not dialysis depend |
| ARF without dialysis | Dx Code with first three digits = ('N17', or 'N19') and POA Code NE ‘Y’ |
| Arrhythmia (includes heart block, atrial fibrillation, or ventricular arrhythmia) | **Heart Block** = Dx Code = ('I440', 'I441', 'I442', 'I444', 'I445', 'I447', 'I450', 'I452', 'I453', 'I454', 'I455', 'I459', 'I4581', or 'I4589') or Any Dx Code with first four digits = (‘I443', 'I446', 'I451')) and POA Code NE 'Y'  **Atrial Fibrillation** = Dx Code with first three digits = ‘I48’ and POA Code NE 'Y'  **Ventricular Arrhythmia** = Dx Code with first three digits = ‘I47’ or ‘I49’ and POA Code NE 'Y' |
| Permanent pacemaker implantation | Px Code = ('0JH604Z', '0JH634Z', '0JH804Z', '0JH834Z', '0JH605Z', '0JH635Z', '0JH805Z', '0JH835Z','0JH606Z', '0JH636Z', '0JH806Z', '0JH836Z', '0JH607Z', '0JH637Z', '0JH807Z', '0JH837Z', or '02HK3NZ') |
| Repeat TAVR | Two different Px Codes = ('02RF37Z', '02RF38Z', '02RF3JZ', '02RF3KZ', '02RF37H', '02RF38H', '02RF3JH', '02RF3KH', '02RF47Z', '02RF48Z', '02RF4JZ', '02RF4KZ', 'X2RF332', or 'X2RF432') |
| SAVR | Px Code = ('X2RF032', '02RF07Z', '02RF08Z', '02RF0KZ' or '02RF0JZ') |
| Vascular complication (includes peripheral vascular complication, cardiac hematoma, cardiac hemorrhage, transfusion, and vascular repair) | **Peripheral Vascular** = Dx Code with first four digits = ('T817') and POA Code NE ‘Y’  **Cardiac Hematoma =** Dx Code with first four digits = ('I974') and POA Code NE ‘Y’  **Cardiac Hemorrhage =** Dx Code with first four digits = ('I976') and POA Code NE ‘y”  **Transfusion =** Px Code with first three digits = ('302')and with sixth digit = ('H', 'J', 'K', 'L', 'M', 'N', 'Q', 'R', 'T', 'V', or 'W')  **Vascular Repair** = Px Code with first three digits = ('03U', '04U', '03Q', or '04Q') |

Dx = diagnostic; Px = procedure
